# Association of leukocyte nadir with complete remission in Indonesian acute myeloid leukemia patients undergoing 7+3 remission induction chemotherapy

**DOI:** 10.12688/f1000research.110320.2

**Published:** 2022-07-01

**Authors:** Dwi Wahyunianto Hadisantoso, Dody Ranuhardy, Wulyo Rajabto, Aulia Rizka, Lyana Setiawan, Ikhwan Rinaldi, Arif Mansjoer, Erni Juwita Nelwan, Hamzah Shatri

**Affiliations:** 1 Department of Internal Medicine, Faculty of Medicine, Universitas Indonesia, Central Jakarta, Greater Jakarta, 10430, Indonesia; 2 Hematology-Medical Oncology, Dharmais Hospital National Cancer Center, West Jakarta, Greater Jakarta, 11420, Indonesia; 3 Department of Internal Medicine, Faculty of Medicine, Dr. Cipto Mangunkusumo National Central Public Hospital, Central Jakarta, Greater Jakarta, 10430, Indonesia; 4 Clinical Pathology, Dharmais Hospital National Cancer Center, West Jakarta, Greater Jakarta, 11420, Indonesia

**Keywords:** acute myeloid leukemia, leukocyte nadir, induction chemotherapy, complete remission, association

## Abstract

**Background**: The 7+3 regimen is still the main choice of remission induction chemotherapy in acute myeloid leukemia (AML). Successfully achieving complete remission (CR) and the time required to achieve it determine patient’s survival. Hence, bone marrow examination on 14 ^th^ day of chemotherapy is recommended to predict CR. However, the examination is invasive and still inaccurate.

**Methods: **A prognostic study with retrospective cohort design was conducted at two central hospitals in Indonesia based on medical record data of AML patients who underwent 7+3 induction chemotherapy from January 1st, 2015, to December 31st, 2019. The association of nadir leukocyte level and the time required to achieve it with CR occurrence was assessed.

**Results: **One hundred and one subjects were recruited with median age 39 years and 55% men. A total of 55.4% subjects achieved CR. Nadir leukocyte level below 200/mcl was the most optimal cut-off point and independently associated with CR (OR 2.48; 95% CI 1.03–5.97) while time required to achieve it was not.

**Conclusions**: The nadir leukocyte level is associated with an increase probability of CR but not for the time required to achieve it in AML patients undergoing 7+3 induction chemotherapy.

## Introduction

Acute myeloid leukemia (AML) is the most common form of acute leukemia in the adult population. ^1^^,^^2^ AML is not a single disease entity, but rather a heterogeneous group of diseases, at least from the clinical picture of blast cell morphology and also at the genetic level which turns out to have a more pronounced prognostic impact. ^3^^–^^7^ This heterogeneity has implications for treatment response and prognosis. ^8^^,^^9^ However, the treatment principles for AML have not changed much. The first goal of curative treatment of AML is always to achieve complete remission through induction chemotherapy whose backbone regimen has not changed since it was first introduced 40 years ago. ^10^^,^^11^

For the purpose of curative treatment in AML, achieving complete remission (CR) as soon as possible after induction chemotherapy is very important because it determines the patient's survival. ^12^ Patients with a treatment response less than CR have lower survival rate than the group of patients who achieve CR. ^4^^,^^13^ The time required to reach CR also carries prognostic significance. Ciftciler reported that patients who achieved CR within 30 days of the start of remission-induced chemotherapy had a better prognosis than patients who required a longer time. ^14^

Therefore, both National Comprehensive Cancer Network (NCCN) and European Society for Medical Oncology (ESMO) recommend bone marrow examination on the 14th day of chemotherapy to predict the occurrence of CR so that reinduction chemotherapy can be carried out earlier if the patient is predicted not to achieve CR. If the bone marrow does not reach a hypoplastic condition, defined as bone marrow cellularity <20% and residual blast cells <5%, it is recommended that the patient be given reinduction chemotherapy immediately. ^9^^,^^15^ However, this examination is invasive and the general condition of the patient during this period is usually very weak with severe pancytopenia which means the procedure still carries some risk for the patient. ^16^ Some reports also show that bone marrow examination is still less accurate in predicting CR because there are some patients who still achieve CR even when their bone marrow examination on day 14 does not show hypoplastic conditions. ^15^^–^^19^

On the other hand, induction chemotherapy also causes cells in the peripheral blood to undergo a nadir and recovery cycle similar to conditions in the bone marrow, especially the leukocyte series. The understanding that the sensitivity of tumor cells to chemotherapy drugs is influenced by the genetic predisposition of the host also supports the idea that this chemotherapy sensitivity is shared by other cells in the individual's body, including leukocytes. ^20^ Pharmacokinetic tests of drugs used for induction chemotherapy has also shown that the level of the drug in leukocytes was directly proportional to its concentration in nucleated cells in the bone marrow. ^21^ This has sparked the idea of the potential use of the leukocyte nadir pattern as a predictor of CR in AML patients undergoing 7+3 remission induction chemotherapy. Examination of peripheral blood leukocyte levels also has several advantages. First, peripheral blood sampling does not have to be done by a trained specialist doctor but can also be done by nurses or laboratory personnel. Second, the examination is also widely available, and the cost is much cheaper than bone marrow examination. Third, from the patient's perspective, peripheral blood examination is more comfortable and causes less anxiety than bone marrow examination.

The aim of this study was to examine the associations of nadir leukocyte level and the time to reach it with the occurrence of CR in AML patients who underwent “7+3” remission induction chemotherapy. The hypothesis of this study was that the nadir leukocyte level and the time to reach it were associated with the occurrence of complete remission in AML patients undergoing 7+3 remission induction chemotherapy.

## Methods

This was a prognostic study with a retrospective cohort design. The research sample was taken by total sampling from the medical record data of patients with a diagnosis of AML who were not acute promyelocytic leukemia (APL) or AML M3 FAB classification who underwent 7+3 induction chemotherapy at Dharmais Hospital National Cancer Center and Dr. Cipto Mangunkusumo National Central Public Hospital during the period from January 1 ^st^, 2015, to December 31 ^st^, 2019. The acceptance criteria for this study were patients aged ≥18 years, diagnosed with AML according to WHO diagnostic criteria (based on at least a bone marrow smear or biopsy and myeloid lineage confirmation from bone marrow aspirate or peripheral blood immunophenotyping), who underwent a first-line remission induction chemotherapy 7+3 regimen, and had never undergone any remission induction chemotherapy before. The criteria for rejection were AML M3 (FAB criteria) or acute promyelocytic leukemia (APL), a myeloblastic crisis phase of chronic myeloid leukemia (CML) or when the required data were not found in the patient's medical record. By estimating the proportion of achieving CR of 60% with margin of error of 5%, a total of 103 subjects were needed for this study. The study was approved by the Universitas Indonesia Ethics Board, approval number KET-603/UN2.F1/ETIK/PPM.00.02/2021. Data were collected from June 15 ^th^ to August 31 ^st^, 2021.

### Research variables

The nadir leukocyte level and the time required to reach it were assessed for their associations to the occurrence of CR during the evaluation of 7+3 remission induction chemotherapy treatment. Treatment evaluations were done when the peripheral blood cells had recovered. The criteria used to define the occurrence of CR during treatment evaluation were in accordance with those established by European LeukemiaNet 2017. ^22^ Factors considered as potential confounders were age, gender, AML subtype, Charlson Comorbidity Index (CCI), history of myelodysplasia syndrome (MDS), history of chemotherapy/radiotherapy, prechemotherapy leukocyte level, myeloblast level at diagnosis, dose intensity of induction chemotherapy agents, occurrence of febrile neutropenia and administration of granulocyte colony-stimulating factor (GCSF). The dose intensity was represented by median cumulative dose, that was the total dose of each chemotherapy drug used in the whole process of induction chemotherapy.

### Statistical analysis

Data processing was carried out using IBM SPSS Statistics version 28 (IBM SPSS Statistics, RRID:SCR_016479). Numerical data were presented as a mean with a standard deviation if the distribution was normal or as a median with a range if the distribution was not normal. Statistical significance testing was carried out according to the characteristics of the data and their objectives. Bivariate testing on nominal data was carried out by using the chi-square test or by using Fisher's exact test as an alternative if the requirements were not met. To see the difference in the mean in two groups with numerical data that had a normal distribution, an unpaired t-test was used, or the alternative Mann-Whitney U test was used instead if the distribution was not normal. The limit of significance (α) was set at 5% in the conclusion of statistical significance.

For data on nadir leukocyte levels and the time required to achieve it in the form of numerical data, the most optimal threshold was sought through the ROC (receiver operating characteristic) curve by assessing sensitivity and specificity values. The power of discrimination of the two variables was measured by the AUC (area under the curve) value. The strength of the association was expressed in terms of relative risk (RR) or odds ratio (OR) with a 95% confidence interval (CI). Variables that had the potential to become confounders were assessed for their relationship with CR occurrence through the bivariate test. When there was a variable that had p-value <0.25 in the bivariate test, this variable would be further analyzed through a multivariate logistic regression test to determine whether the variables acted as a confounder or not by looking at the changes in the OR it caused. A variable was defined as a confounder when it changed the OR (ΔOR) >10%.

## Results

**Figure 1.**
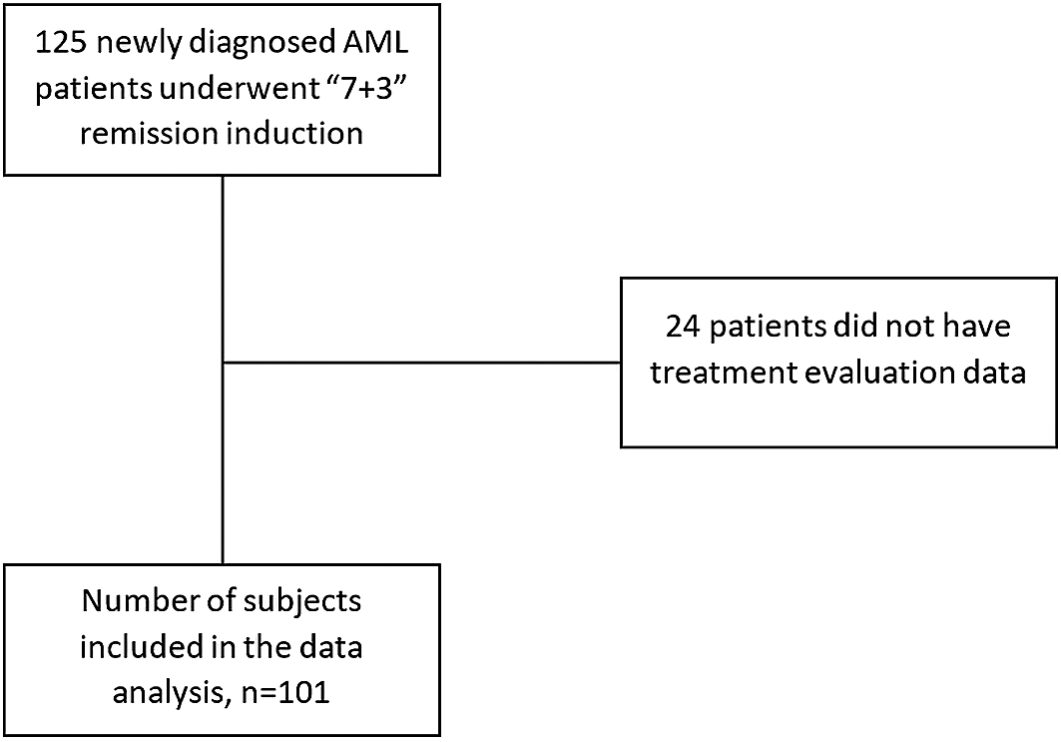
Research subject recruitment flow-chart.

We found 125 patients with newly diagnosed AML non-APL who underwent 7+3 remission induction chemotherapy in the period from January 1 ^st^, 2015, to December 31 ^st^, 2019. Twenty-four patients were excluded because they died while undergoing the chemotherapy hence, they did not have treatment outcome data. In the end, there were 101 subjects whose data could be analyzed. The recruitment process of research subjects is shown in Figure 1.

The median age of the research subjects was 39 years (range 18–66 years). Male subjects were slightly more common than females with a ratio of 1.2, while the most common subtype was AML M2 (48.5%). The majority of patients had no comorbidities, no history of MDS, and none had a history of chemotherapy/radiotherapy. No patient had cytogenetics nor day-14 bone marrow examination data. Median daily doses of chemotherapy agents used, daunorubicin and cytarabine, in the 7+3 protocol were 50 and 102 mg/m ^2^/day, respectively, while the median cumulative dose of daunorubicin and cytarabine were 150 and 711 mg/m ^2^, respectively, as shown in Table 1. Subjects diagnosed in 2018 and thereafter had significantly higher median daily dose of daunorubicin (58 vs 47 mg/m ^2^/day; p<0.01) and cytarabine (168 vs 100 mg/m ^2^/day; p=0.02) than those diagnosed before 2018 (See *Underlying data*). ^23^

**Table 1.** Characteristics of research subjects.

Characteristics	N (%) (total, n = 101)	Characteristics	N (%) (total, n = 101)
Age (year)		History of Chemo/Radiotherapy	
Median, range	39 (18–66)	No	101 (100)
		Yes	0 (0)
Sex			
Male	55 (54.5)	Daunorubicin daily dose (mg/m ^2^/day)	
Female	46 (45.5)	Median, range	50 (33–67)
AML Subtypes		Daunorubicin cumulative dose (mg/m ^2^)	
M0	-	Median, range	150 (99–202)
M1	11 (1.9)		
M2	49 (48.5)	Cytarabine daily dose (mg/m ^2^/day)	
M4	26 (25.7)	Median, range	102 (66–222)
M5	7 (6.9)		
M6	-	Cytarabine cumulative dose (mg/m ^2^)	
M7	-	Median, range	711 (460–1,544)
NOS	8 (7.9)		
		GCSF usage	
Pre-Chemo Leukocyte Level (/mcl)		Before leukocyte nadir	41 (40.6)
Median, range	13,600 (660–314,280)	After leukocyte nadir	24 (23.8)
		Did not use	36 (35.6)
Blast level at diagnosis (%)			
Mean *,* SD	59.32 (18.78)	Leukocyte nadir level (/mcl)	
		Median, range	230 (40–2,020)
*Charlson comorbidity index*			
0	91 (90.1)	Time to leukocyte nadir (days)	
≥1	10 (9.9)	Median, range	14 (4–35)
History of MDS		Complete remission	
No	93 (92.1)	Yes	56 (55.4)
Yes	8 (7.9)	No	45 (44.6)

### Treatment results of 7+3 remission induction chemotherapy

A total of 56 subjects (55.4%) achieved CR. There was a difference in the median nadir leukocyte level between the group of subjects who managed to achieve CR (190/mcl; range 40–940/mcl) and the group of subjects who did not (250/mcl; range 70–2,020/mcl; p = 0.02). However, the median number of days required to reach the leukocyte nadir did not differ between the groups that achieved CR (13.5 days; range 4–35 days) and those who failed (14 days; range 5–24 days; p = 0.50). Therefore, in the next analysis, only nadir leukocyte level as the independent variable was examined and its relation to CR occurrence as the dependent variable.

### Determining the threshold for nadir leukocyte levels

**Figure 2.**
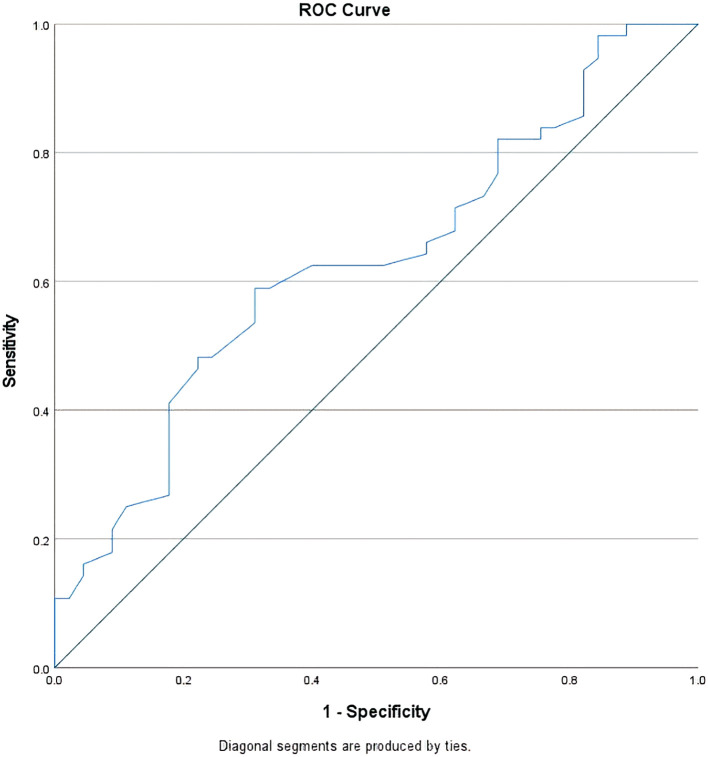
ROC curve of nadir leukocyte levels and CR occurrence during treatment evaluation. Diagonal segments are produced by ties.

To find the optimal threshold of the nadir leukocyte level that has a prognostic value to CR occurrence, the specificity and sensitivity of each value of nadir leukocyte level in the occurrence of CR during treatment evaluation were analyzed using the ROC (receiver operating characteristic) curve as shown in Figure 2.

From this curve, the AUC value was 0.63 (95% CI 0.52–0.74) and the most optimal cut-off point for the nadir leukocyte level was 200/mcl (73% specificity, 50% sensitivity). Based on this cut-off point, there were 40 subjects (39.6%) whose nadir leukocyte level was <200/mcl with the RR achieving complete remission of 1.52 (95% CI 1.09–2.14) compared to the group whose nadir leukocyte level was higher. In the subgroup analysis, the subject groups that reached nadir leukocytes level of <200/mcl had a higher CR proportion compared to the groups that had higher nadir leukocyte level even though not all of them reached statistical significance (see *Underlying data*). ^23^ In addition, along with the lower cut-off value of the nadir leukocyte level, the proportion of subjects with CR became higher (see *Underlying data*). ^23^

### Association between nadir leukocyte levels and complete remission occurrence during treatment evaluation

After dividing subjects into two groups based on the 200/mcl leukocyte level threshold, bivariate analysis was performed along with the other variables that had the potential to be confounders to the occurrence of CR as the dependent variable as shown in Table 2. For the GCSF administration variable, only GCSF given to subjects before the leukocyte nadir was reached was considered to be a potential confounder for the relationship between the nadir leukocyte level and the occurrence of CR. A total of 41 subjects (40.6%) received GCSF before the nadir leukocyte level was reached.

**Table 2.** Bivariate test results for each independent variable with the dependent variable.

Variables	CR (n,%) (total, n = 101) **		*p-value*	Variables	CR (n,%) (total, n = 101) **		*p-value*
	Yes	No			Yes	No	
Nadir leukocyte level (/mcl)			0.02 *	History of Chemo/ Radiotherapy			n.a.
<0.20	28 (70.0)	12 (30.0)		No	56 (55.4)	45 (44.6)	
≥0.20	28 (45.9)	33(54.1)		Yes	0 (0)	0 (0)	
Age (year)			0.75	History of MDS			0.75
<60	52 (55.9)	41 (44.1)		No	52 (55.9)	41 (44.1)	
≥60	4 (50.0)	4 (50.0)		Yes	4 (50)	4 (50)	
CCI			0.33	Daunorubicin cumulative dose (mg/m ^2^)			0.88
0	49 (53.8)	42 (46.2)		≥150	29 (54.7)	24 (45.3)	
≥1	7 (70.0)	3(30.0)		<150	27 (56.3)	21 (43.8)	
AML Subtypes			0.82	Cytarabine cumulative dose (mg/m ^2^)			0.10 *
M1	6 (54.5)	5 (45.5)		≥711	34 (63.0)	20 (37.0)	
M2	30 (61.2)	19 (38.8)		<711	22 (46.8)	25 (53.2)	
M4	13 (50.0)	13 (50.0)					
M5	4 (57.1)	3 (42.9)		GCSF administration prior to leukocyte nadir			0.08 *
				No	29 (48.3)	31 (51.7)	
Blast level at diagnosis (%)			0.17 *	Yes	27 (65.9)	14 (34.1)	
*Mean,* SD	57.02 (±17.55)	62.20 (±20.03)					
				Febrile neutropenia			0.93
Pre-Chemo Leukocyte Level (/mcl)			0.37	No	17 (54.8)	14 (45.2)	
Median, range	11,500 (660–187,910)	21,360 (1,620–314,280)		Yes	39 (55.7)	31 (44.3)	

* variable with p < 0.25.

** except AML subtypes (n = 93).

From the results of the bivariate test, there were three variables that had p < 0.25 other than the nadir leukocyte level (p = 0.02), those were administration of GCSF before the leukocyte nadir (p = 0.08), cytarabine cumulative dose (p = 0.10) and myeloblast level at diagnosis (p = 0.17). To see the magnitude of the effect of nadir leukocyte level on the achievement of CR and to determine whether the other variables would act as confounders, a multivariate test was carried out using the logistic regression method. The crude OR was 2.75 (95% CI 1.12–6.39) while fully adjusted OR 2.48 (95% CI 1.03–5.97). None of the variables presumed as confounders changed OR more than 10% as shown in Table 3.

**Table 3.** Multivariate analysis results.

Variables	OR (95% CI)	ΔOR
*Crude* OR:		
Nadir leukocyte level	2.75 (1.18–6.39)	
*Adjusted* OR:		
+ GCSF administration	2.55 (1.09–6.00)	7.71%
+ Cytarabine cumulative dose	2.54 (1.06–6.08)	0.39%
+ Myeloblast level at diagnosis	2.48 (1.03–5.97)	2.42%

## Discussion

Out of 125 patients who underwent “7+3” remission induction chemotherapy, 24 subjects (19.2%) did not have treatment outcome data because they died before treatment evaluation and had to be excluded. Deaths in this period were grouped into treatment related mortality (TRM). This figure is not much different from that obtained by Kayal in India (16.9%) but definitely higher than that obtained by Gbadamosi in the US (6.9%). ^24^^,^^25^ The results of this study indicate that there is still a gap in the quality of AML treatment between developing and developed countries. ^2^^,^^5^^,^^26^^,^^27^

The median age of this study (39 years) differs from the median age of AML cases in the general population (68 years) because the subjects taken were only AML patients who underwent intensive treatment and successfully completed it. Of all subjects whose treatment results could be evaluated, 55.4% managed to achieve CR. Again, this figure is not much different from that obtained by Kayal in India (52.8%) but lower than Gbadamosi in the US (62%). ^24^^,^^25^ Gbadamosi’s higher CR figure was probably due to the fact that the US treatment facilities are better than Indonesia and India. ^2^^,^^10^^,^^28^ Another factor that might play a role in this low rate was the low dose of chemotherapy drugs used in this study. Infection control barriers and TRM rates in developing countries might encourage clinicians to use lower limits of the recommended dose. Yet, the increasing median daily dose of both chemotherapy agents for subjects diagnosed in 2018 thereafter compared to those diagnosed earlier, especially for daunorubicin, might indicate that the treating physicians needed time to absorb newer guidelines which recommend higher dose of daunorubicin (60–90 mg/m ^2^/day) than the previous (45–60 mg/m ^2^/day), as more and more physicians implemented higher dose of daunorubicin with increasing year.

There was a difference in median nadir leukocyte levels in the group that managed to achieve CR compared to the group that did not, but the same could not be said on the number of days required to reach the leukocyte nadir. The data that showed a uniform median number of days needed to reach the leukocyte nadir between the two groups (i.e. 14 days) was in accordance with the day recommended by ESMO and NCCN guidelines to perform a bone marrow examination. ^9^ This result is similar to that reported by Marras where the time required for nadir leukocyte level to be reached was also 12 days for both *responder* and *non-responder* groups. ^29^ This reinforces the premise that the nadir pattern of leukocytes in the peripheral blood is similar to the pattern of hypoplasia in the bone marrow.

However, this result is different from that obtained by Han who reported that there was a difference in the proportion of subjects who achieved CR between the groups who needed more than 10 days to reach the leukocyte nadir and those who needed less. ^17^ These different results are probably due to differences in the characteristics of the subjects in that Han's study only recruited subjects over 55 years of age as well as differences in the AML treatment technique used. Old age is associated with a decrease in the activity of hematopoietic stem cells in the bone marrow and the mobilization of PMN cells from the bone marrow to the peripheral blood. ^30^^,^^31^ The most striking difference in treatment technique was that Han allowed his subjects to undergo reinduction chemotherapy if the results of the bone marrow examination on day 14 still contained *blast* cell residues >5% while none of our subjects received reinduction chemotherapy before treatment evaluation.

This study found that the threshold for the most optimal nadir leukocyte level that had a prognostic value on the occurrence of CR during treatment evaluation was 200/mcl with an *acceptable* discriminant power (AUC 0.63) in identifying subjects who would achieve CR and those who would not (p = 0.02). ^32^ From the RR calculation, the number of subjects whose nadir leukocyte levels was <200/mcl managed to achieve CR 1.52 times more than subjects with higher nadir leukocyte levels. This association was independent based on the results of the multivariate analysis using the logistic regression method with fully adjusted OR 2.48 (95% CI 1.03–5.97). By looking at the OR changes from each step of the logistic regression analysis, none of them made OR changes >10%. Hence, it could be concluded that there were no variables acting as confounders in this study.

The association between nadir leukocyte level to the achievement of CR was then analyzed to see if it met the principles of causality by Sir Austin Bradford Hill. ^33^ The first principle is the temporal relationship, in which the independent variable in terms of time must precede the dependent variable. In this case, the nadir leukocyte level as an independent variable always preceded the occurrence of CR.

The second principle is an association that emphasizes the strength of the relationship between the independent variable and the dependent variable where the stronger the relationship between the variables, the more probable the concept of causality. From this study, the strength of the association was represented by a fully adjusted OR of 2.48 which is statistically significant.

The third principle is dose-dependent, if the size of the dependent variable changes along with the change in size of the independent variable, then a causal relationship becomes more likely. For the group of subjects with nadir leukocyte levels below 300/mcl, 200/mcl, and 100/mcl, the proportions of achieving CR were 58.1%, 70%, and 81.8% respectively (see *Underlying data*). ^23^ The proportion of the occurrence of CR was getting higher as the threshold for the nadir leukocyte level lower.

The fourth principle is consistency, the relationship between the independent variable and the dependent variable remains consistent when applied to different subjects or observations. In the group of male and female subjects or the elderly and non-elderly, the group of subjects with a nadir leukocyte level <200/mcl consistently achieved more CR than the group of subjects with a higher nadir leukocyte level, although the differences were not always statistically significant (see *Underlying data*). ^23^

The fifth principle is coherence in which research results do not conflict with existing knowledge about the disease. Kinetics of leukocyte has long been used as an indicator of bone marrow recovery, which is characterized by absolute neutrophil count (ANC) levels reaching above 500/mcl after nadir. ^29^ This shows that the concept of low nadir leukocyte levels as a surrogate marker of the degree of bone marrow hypoplasia associated with CR does not conflict with existing knowledge about AML.

The sixth principle is biological plausibility where research results can be explained by existing theories. The degree of decrease in peripheral blood leukocyte levels due to chemotherapy exposure is considered to be comparable to what happened in blast leukemia cells, and it has been demonstrated, at least in breast cancer and lung cancer, that the degree of leukopenia is associated with response to chemotherapy. ^34^^,^^35^

The seventh principle is the suitability of the results with other studies. To our knowledge, there have been no other studies reporting similar results, thus the nadir leukocyte nadir level of 200/mcl is the novelty of this study. Therefore, this principle cannot be determined at this time, and further research is needed to confirm the results found in this study. Thus, it has been shown that the results of this study are in accordance with most of the causality principles introduced by Sir Austin Bradford Hill but still require further research to confirm this relationship.

This study showed that lower nadir leukocyte levels (<200/mcl) were associated with higher proportion of subjects achieving CR than the group with higher nadir leukocyte levels. A lower level of leukocyte nadir has long been associated with the higher exposure level of chemotherapy drugs in the bone marrow and blood cells and is expected to describe drug exposure to tumor cells. ^20^ It is expected that the lower level of leukocyte nadir is related to the higher amount of eradicated leukemic blast cells in the patient's bone marrow. However, the role of nadir leukocyte level on CR occurrence during treatment evaluation in this study was only as strong as 63% which was symbolized by the AUC value of the ROC curve thus opening up the possibility that other variables might play roles in AML patients who achieved CR. Hence, the cut-off point for the nadir leukocyte levels reported in this study still cannot be directly applied to daily clinical practice. A predictive model that involves other variables is needed to improve the performance of predicting CR occurrence.

### Research limitations

The main limitation of this study as in other studies with retrospective design is the impossibility in controlling the variables studied. Although regimens and protocols of chemotherapy for remission induction 7+3 are standard, other treatments given to patients might vary widely between clinicians and might have prognostic impacts on patients that were not possible to include in the analysis. The sample of this study also involved only a few elderly subjects, subjects with comorbidities, or subjects with secondary AML so that the influence of these variables might be less visible in this study. In addition, the absence of cytogenetic profile data in the study sample made it impossible to assess the role of leukemia blast cell characteristics, especially in terms of the sensitivity of AML to treatment. Yet, the limited facilities for cytogenetics and molecular examinations in developing countries, including Indonesia, make simpler alternative tests (e.g., peripheral blood leukocyte levels) in providing information about disease behavior even more necessary to determine the best treatment strategy in AML patients. However, the results of this study still need to be confirmed in the patient group based on the risk of AML through cytogenetics in follow-up studies.

## Conclusions

AML patients undergoing “7+3” remission induction chemotherapy who managed to achieve nadir leukocyte level <200/mcl is associated with an increased probability of CR. Time to reach the nadir leukocyte level does not have an association with the occurrence of CR.

## Data availability

### Underlying data

Mendeley Data: Underlying data for ‘Association of leukocyte nadir with complete remission in Indonesian acute myeloid leukemia patients undergoing 7+3 remission induction chemotherapy’. http://doi.org/10.17632/xfx39znwzp.3 ^23^

This project contains the following underlying data:

• Data file 1: Dataset file ver 3 published.sav
• Data file 2: Supplementary Data – Changes in Proportion of CR Occurrence Along with Changes in the Nadir Leukocyte Level Threshold.docx
• Data file 3: Supplementary Data – Association of Nadir Leukocyte Level Less Than 200mcl with CR Occurrence in Several Subjects Groups.docx
• Data file 4: Supplementary Data – The Median Daily Doses of Chemotherapy Agents by Time Periods of Diagnosis.docx

## Reporting guidelines

Mendeley Data: STROBE checklist for ‘Association of leukocyte nadir with complete remission in Indonesian acute myeloid leukemia patients undergoing 7+3 remission induction chemotherapy’. http://doi.org/10.17632/xfx39znwzp.3 ^23^

Data are available under the terms of the Creative Commons Attribution 4.0 International license (CC-BY 4.0)

## Consent

Written informed consent for publication of the patients’ details was obtained from the patients.
