# Supplementary material for: Association of leukocyte nadir with complete remission in Indonesian acute myeloid leukemia patients undergoing 7+3 remission induction chemotherapy
Source: F1000Res. 2022 Jul 1;11:495. Originally published 2022 May 5. [Version 2] doi: 10.12688/f1000research.110320.2 (PMC9194516; doi:10.12688/f1000research.110320.2)
Supplement: Peer review history

# Peer review: Reviewer response for version 2

**DOI:** 10.5256/f1000research.135572.r142927

**Published:** 2022-08-31

**Authors:** Shinta Wardani

**Affiliations:** 1 Division of Hematology and Oncology, Department of Internal Medicine, Faculty of Medicine, Universitas Brawijaya, Malang, Indonesia

I found the responses from the authors were sufficient to answer all of my questions. The manuscript is acceptable without any further reservation.

Is the work clearly and accurately presented and does it cite the current literature?

Partly

If applicable, is the statistical analysis and its interpretation appropriate?

I cannot comment. A qualified statistician is required.

Are all the source data underlying the results available to ensure full reproducibility?

Partly

Is the study design appropriate and is the work technically sound?

Yes

Are the conclusions drawn adequately supported by the results?

Yes

Are sufficient details of methods and analysis provided to allow replication by others?

Yes

Reviewer Expertise:

NA

I confirm that I have read this submission and believe that I have an appropriate level of expertise to confirm that it is of an acceptable scientific standard.

# Peer review: Reviewer response for version 1

**DOI:** 10.5256/f1000research.121914.r137008

**Published:** 2022-06-13

**Authors:** Smita Kayal

**Affiliations:** 1 Department of Medical Oncology, Jawaharlal Institute of Postgraduate Medical Education and Research, Puducherry, Puducherry, India

This retrospective study on role of leucocyte nadir in predicting remission outcomes of intensive induction therapy in AML (acute myeloid leukemia) is an important work, especially in resource limited limited settings. It is well written with lucid discussion. 

- Limitations of absence of cytogenetic data, and other aspects are mentioned. However, a comment should be added on lower average dose of daunorubicin used in the study and reason for it. 
- In table 2, n to be added in column headings.
- Whether other cell components (neutrophil or lymphocyte) were also analyzed for their predictive value? 

Is the work clearly and accurately presented and does it cite the current literature?

Yes

If applicable, is the statistical analysis and its interpretation appropriate?

Yes

Are all the source data underlying the results available to ensure full reproducibility?

Yes

Is the study design appropriate and is the work technically sound?

Yes

Are the conclusions drawn adequately supported by the results?

Yes

Are sufficient details of methods and analysis provided to allow replication by others?

Partly

Reviewer Expertise:

hemato-oncology, transplant, supportive care

I confirm that I have read this submission and believe that I have an appropriate level of expertise to confirm that it is of an acceptable scientific standard.

# Response

**Published:** 2022-06-26

**Authors:** Dwi Wahyunianto Hadisantoso

**Affiliations:** University of Indonesia, Indonesia

Dear Dr. Smita Kayal, we are so delighted for your valuable response and comments. Here is our response:

1. We have added some conditions that might explain the lower median daily dose of daunorubicin in this study in the last two sentences of second paragraph in “Discussion” section. We think of the higher TRM (treatment related mortality) rates in developing countries and time needed by treating clinicians to absorb newer guidelines might have significant contributions.
2. We have added “n” as total subjects in column headings of Table 2.
3. We did not analyze the counts of leukocyte differential because as the lower the level of leukocyte, the more unreliable the differential count result would become as the CV (coefficient of variance) would increase dramatically. Thus, we were afraid this could lead to a significant bias, especially for a retrospective study using secondary data. This effect also happens on leukocyte (as total white blood cells) count but far less than on its differential. The other reason is not all subjects had daily leukocyte differential count data.

# Peer review: Reviewer response for version 1

**DOI:** 10.5256/f1000research.121914.r137004

**Published:** 2022-06-07

**Authors:** Hyewon Lee

**Affiliations:** 1 Center for Hematologic Malignancy, National Cancer Center, Goyang, South Korea

The aim of the study is to investigate the correlation between nadir leukopenia and complete remission in patients treated with conventional induction therapy for AML (acute myeloid leukemia). It was a retrospective study, which included 101 patients who could recover from nadir and underwent response evaluation.

 The binominal analyses and ROC (receiver operating characteristic) curve were adopted to explore any association between the two variables in this study. For this, the outcome should be defined as two groups, such as ’CR achieved’ or ‘CR not achieved’. The authors excluded 24 patients from initial cohort (n=125), because they died early before response evaluation. However, this may be a bias underestimating the proportion of CR (complete remission) group, because early death might be associated with treatment toxicity, low WBC count and prolonged nadir. This may be one of the reason why CR rate is relatively low in developing countries compared to developed countries.

 In addition, nadir leukopenia is a result of intensive chemotherapy. However, the median dose of daunorubicin in this study was 50mg/m2/day, which is lower than standard dose. It should be considered when the authors analyze nadir leukocyte count and the outcome of chemotherapy. Furthermore, dose intensity should be described median cumulative dose of entire induction therapy. Because lower dose intensity is supposed to correlate to relatively higher nadir leukocyte count and subsequent induction failure, it might mislead the authors to conclude that nadir leukocyte count itself has an impact on complete remission.

 To avoid this misinterpretation, multivariate analysis for achieving CR should be performed with more variables such as age, cumulative dose intensity and cytogenetic risk group of AML, in addition to variables which were included in the presented study. Then comprehensive discussion on the results should be done, focusing on possible reasons why it was shown like that.

 If there is no available data of AML biology, it should be described as a limitation of this study in discussion session.

Is the work clearly and accurately presented and does it cite the current literature?

Yes

If applicable, is the statistical analysis and its interpretation appropriate?

Partly

Are all the source data underlying the results available to ensure full reproducibility?

Yes

Is the study design appropriate and is the work technically sound?

Partly

Are the conclusions drawn adequately supported by the results?

Partly

Are sufficient details of methods and analysis provided to allow replication by others?

Yes

Reviewer Expertise:

Hematooncology

I confirm that I have read this submission and believe that I have an appropriate level of expertise to confirm that it is of an acceptable scientific standard, however I have significant reservations, as outlined above.

# Response

**Published:** 2022-06-26

**Authors:** Dwi Wahyunianto Hadisantoso

**Affiliations:** University of Indonesia, Indonesia

Dear Dr. Hyewon Lee, we are so delighted to have your valuable response and comments. Here is our response:

1. We agree that subjects who underwent treatment related mortality (TRM) can be a bias for the reported proportion of complete remission (CR). On the same reason, we also agree that higher rate of TRM in developing countries can be the reason why the rate of CR is lower compared to developed countries. Nevertheless, we had to exclude this group of subjects from analysis since the treatment response could not be determined. Follow up study to elucidate the characteristics of subjects who underwent TRM after induction chemotherapy might give valuable information of these premises and help to reduce its rate.
2. We agree that lower dose of chemotherapy used in this study could have an impact in the outcome of induction chemotherapy, as we wrote in the last two sentences of the second paragraph in “Discussion” section. As this study had retrospective design that recruited subjects from January 2015 to December 2019 we think another reason that could explain the lower dose of daunorubicin is the possibility that clinicians need time to move on from the previous guidelines that recommended daunorubicin dose range between 45 – 60 mg/m2/day to the current 60 – 90 mg/m2/day as there is significant difference of median daily dose of daunorubicin for subject who were diagnosed before 2018 and 2018 afterward (47 vs 58 mg/m2/day, respectively; p=0,004). More and more clinicians were implementing higher dose of daunorubicin with increasing year to the dose range recommended by current guidelines.
3. We agree to use “median cumulative dose” as it seems more appropriate to describe the chemotherapy dose intensity (and consequently used in analysis) rather than the median of daily dose, yet we still report the median daily dose of each drug in the article as it is easier to compare them to the recommended daily doses provided by the AML guidelines. Nevertheless, these changes did not change the conclusion of the analysis, there was still no significant association between the median cumulative dose of daunorubicin nor cytarabine with the occurrence of CR (p=0.88 for daunorubicin and p=0.10 for cytarabine). There was indeed a significant association between the subjects who had higher cumulative dose of daunorubicin (≥150 mg/m2) with lower leucocyte nadir level (<200/mcl) (RR=2.11; CI 95% 1.22 – 3.67, p<0.01) but not for cytarabine cumulative dose with leukocyte nadir level (p=0.80). This interesting result may warrant follow-up study to further explore the relationship between chemotherapy dose intensity and nadir of leukocyte.
4. This study aimed to explore the relationship between leukocyte nadir with CR occurrence and some variables that were thought to potentially act as confounders were also analyzed in two steps manner of analysis as we wrote in the “Methods” section and “Statistical analysis” subsection. Those variables were age, gender, AML subtype, Charlson Comorbidity Index (CCI), history of myelodysplasia syndrome (MDS), history of chemotherapy/radiotherapy, prechemotherapy leukocyte level, bone marrow myeloblast cell level at diagnosis, dose intensity of chemotherapy agents, occurrence of febrile neutropenia and administration of granulocyte colony-stimulating factor (GCSF).
  For each of these variables we initially did bivariate analysis to identify the significancy of its relationship to the CR occurrence as the dependent variable. Only variables with p<0.25 from bivariate analysis (GCSF administration, cytarabine cumulative dose, and myeloblast level at diagnosis, as shown in Table 2) were considered “significant” then further analyzed in multivariate analysis to be determined whether they act as confounders in the relationship of leukocyte nadir level (as independent variable) and CR occurrence (as dependent variable) or not. This is to explain that the study already considered variables suggested by the reviewer as potential confounders other than cytogenetic risk group, which the latter had been written at the “Research limitations” section.

# Peer review: Reviewer response for version 1

**DOI:** 10.5256/f1000research.121914.r137006

**Published:** 2022-05-27

**Authors:** Shinta Wardani

**Affiliations:** 1 Division of Hematology and Oncology, Department of Internal Medicine, Faculty of Medicine, Universitas Brawijaya, Malang, Indonesia

This research idea is very original and applicable, especially in countries with limited resources.

 The golden standard for assessing hypoplasia in AML (acute myeloid leukemia) patients after induction therapies bone marrow aspiration, with criteria for bone marrow cellularity <20% and residual blast <5%, which are also a criteria for complete remission. If the author wants to replace the bone marrow aspiration with the nadir leucocyte count, then my question is, has this nadir leucocyte count been compared with the hypoplastic condition through the golden standard bone marrow aspiration?

 On page 8 of the last paragraph, how do the authors show that a low nadir leucocyte count is a surrogate marker of the degree of bone marrow hypoplasia?

 I need your comment, the number of blast cells in the periphery is not needed in assessing bone marrow hypoplasia? Why is it only the nadir leucocyte that is used as a surrogate marker?

 I want to confirm the number of subjects who took part in this study, because the abstract mentioned 101 subjects, but in the method on the full paper 103 patients.

Is the work clearly and accurately presented and does it cite the current literature?

Partly

If applicable, is the statistical analysis and its interpretation appropriate?

I cannot comment. A qualified statistician is required.

Are all the source data underlying the results available to ensure full reproducibility?

Partly

Is the study design appropriate and is the work technically sound?

Yes

Are the conclusions drawn adequately supported by the results?

Yes

Are sufficient details of methods and analysis provided to allow replication by others?

Yes

Reviewer Expertise:

Hematology and medical oncologist

I confirm that I have read this submission and believe that I have an appropriate level of expertise to confirm that it is of an acceptable scientific standard, however I have significant reservations, as outlined above.

# Response

**Published:** 2022-06-05

**Authors:** Dwi Wahyunianto Hadisantoso

**Affiliations:** University of Indonesia, Indonesia

Dear Dr. Shinta Wardani, we would like to thank you for your valuable comments and questions. Here is our response:

1. We need to clarify that the goal of this research is to elucidate the association between leukocyte nadir and complete remission (CR) occurrence in acute myeloid leukemia (AML) patients undergoing 7+3 remission induction based on some evidence that day-14 bone marrow examination (BME), as the gold standard for predicting CR, still has some limitations and inaccuracy.
  Hence, the result of this research is not meant to replace the BME with leukocyte nadir count, yet the latter might give some information for the treating clinicians in predicting CR occurrence irrespective of whether the day-14 BME was done or not to the patient. In this retrospective research we have stated in the article that none of our subjects underwent day-14 BME.
2. Since none of our subjects underwent day-14 BME, we could not directly analyze leukocyte nadir as a surrogate marker of bone marrow hypoplasia. Yet, the study results do not contradict the concept of the pattern of leukocyte nadir in the peripheral blood is similar to the hypoplasia pattern of nucleated cells in the bone marrow. The median time needed to achieve the nadir leukocyte level was equal for both groups (CR group and non-CR group) that were 14 days. This result is in-line with Marras et al (citation no. 29) who also found 12 days as uniform median time needed to achieve nadir leukocyte level for both responder and non-responder groups. These numbers of days are so similar to the bone marrow hypoplasia period, as the ultimate reason why the BME is recommended to be done on day-14 of induction chemotherapy. The study result that showed subjects who succeeded in achieving nadir leukocyte level less than 200/mcl had higher probability in achieving CR than who did not (RR 1.52; CI 95% 1.09 – 2.14) is analogous to the well-accepted paradigm that subjects who succeed achieving bone marrow hypoplasia in day-14 BME are predicted to achieve CR on treatment evaluation. Another concept based on association between leukocyte in peripheral blood and hematopoietic cells in bone marrow has also been used for long, as level of absolute neutrophile count (ANC), one of the leukocyte components, rising above 500/mcl from previously nadir level marks the recovery of the bone marrow.
3. Although we are aware some previous studies have showed correlation between peripheral blast clearance with treatment response, yet we decided to not include it in analysis. Not all AML patients have peripheral blast when they are diagnosed. In this study, there were 8 subjects (7.9%) whose peripheral blast level was 0% and up to 21% of the sample had very few peripheral blast count (less than 5%) before treatment. This condition would make analysis of peripheral blast clearance to treatment response for this group of subjects become impossible and they were always excluded in such studies. Thus, we choose leukocyte nadir as independent variable since it is a universal event that can be analyzed in *all* AML patients undergoing 7+3 induction chemotherapy.
4. From statistic sample size calculation 103 subjects were needed (as we wrote in Method section), yet the study found 101 subjects eligible for analysis (as we wrote in Results section), thus this study sample comprised of 101 subjects.
